# Colorectal Cancer Screening and Surveillance for Non-Hereditary High-Risk Groups—Is It Time for a Re-Think?

**DOI:** 10.1007/s11938-020-00317-8

**Published:** 2021-01-05

**Authors:** James S. Hampton, Linda Sharp, Dawn Craig, Colin J. Rees

**Affiliations:** 1 Department of Gastroenterology, South Tyneside and Sunderland NHS Foundation Trust, South Shields, UK; 2 Population Health Sciences Institute, Newcastle University Centre for Cancer, Faculty of Medical Sciences, Newcastle University, Paul O’Gorman Building, Newcastle Upon Tyne, NE2 4HH UK

**Keywords:** Screening, Colorectal cancer, Surveillance, High risk

## Abstract

**Purpose of review:**

Colorectal cancer (CRC) is the second most common cause of cancer death worldwide, killing approximately 900,000 people each year. An individual’s risk of developing CRC is multi-factorial with known risk factors including increasing age, male sex, family history of CRC and raised body mass index. Population-based screening programmes for CRC exist in many countries, and in the United Kingdom (UK), screening is performed through the NHS Bowel Cancer Screening Programme (BCSP). Screening programmes offer a population-based approach for those at “average risk”, and do not typically offer enhanced screening for groups at increased risk. In the UK, such patients are managed via non-screening symptomatic services but in a non-systematic way.

**Recent findings:**

There is growing evidence that conditions such as cystic fibrosis and a history of childhood cancer are associated with higher risk of CRC, and surveillance of these groups is advocated by some organizations; however, national recommendations do not exist in most countries.

**Summary:**

We review the evidence for screening “high risk” groups not covered within most guidelines and discuss health economic issues requiring consideration acknowledging that the demand on colonoscopy services is already overwhelming.

## Background

Colorectal cancer (CRC) is the second most common cause of cancer in Europe and accounts for nearly 1.9 million incident cases and almost 900,000 deaths per year worldwide [1, 2]. The overwhelming majority of CRCs develop from pre-cancerous adenomas and serrated polyps [3–5].

Colonoscopy is the preferred diagnostic method for CRC; as well as allowing direct visualization of the bowel mucosa to identify abnormalities and take samples, it provides the opportunity to perform therapeutics such as polypectomy to remove potentially pre-cancerous lesions [6]. Colonoscopy is usually performed based upon patient symptoms, as part of population-based screening programmes (as a primary screening test or as a diagnostic test following initial stool blood testing), in people considered “high risk” due to genetic or other factors placing them at increased risk or as a surveillance procedure following a previous abnormal colonoscopy or CRC.

From a patient perspective, colonoscopy requires a hospital visit, can be associated with considerable anxiety and is invasive [7–9]. It has associated risks including bleeding and perforation; therefore, it is vitally important that the benefit of the procedure outweighs any risks [10]. The benefits and risks need to be considered on both an individual and population level and are of particular importance when considering screening and surveillance of asymptomatic populations. Furthermore, the demand on endoscopy services is overwhelming. For example, more than 675,000 colonoscopies are performed annually in the United Kingdom (UK), and services are struggling to cope [11, 12]. The COVID-19 pandemic has significantly reduced endoscopy capacity, and this will have a significant impact upon timely diagnosis and outcomes of cancer [13, 14]. It therefore becomes imperative that colonoscopy resources are prioritized in a manner where they are likely to realize the most benefit, thus ensuring that service capacity is used to maximize health benefits.

In the UK, the lifetime risk of developing CRC is 7% for males and 6% for females [15]. As well as age and sex, established risk factors for CRC include family history, lack of physical activity, excess body weight, aspects of diet, smoking and the presence of certain underlying medical conditions [16–20].

Population-based screening programmes exist in many countries, and in England, screening is performed via the NHS Bowel Cancer Screening Programme (BCSP) [21]. The BCSP provides screening based on faecal immunochemical testing (FIT) in people aged 60–74 years. As in most countries, age is currently the only risk factor considered in this programme with research being undertaken to establish whether screening could, or should, be adapted based upon other risk factors. This article does not review the evidence for population-based screening programmes.

Population-based screening programmes generally offers a protocol appropriate for people at “average risk” of disease. Most programmes do not screen high-risk groups specifically, and in the UK, these are managed through “symptomatic” services. The British Society of Gastroenterology (BSG) produced guidelines in 2010 regarding screening and surveillance in moderate- to high-risk groups [22]. Specific guidance related to surveillance post-polypectomy and post-CRC resection along with the management of hereditary cancers has recently been published [23, 24].

In addition to the patient groups covered in the above guidelines, there are other conditions such as cystic fibrosis, a history of childhood cancer, exposure to ionizing radiation and non-alcoholic fatty liver disease (NAFLD) where there is some evidence of a higher risk of developing CRC [25–28]. Some advocacy groups support screening in these individuals; however, international recommendations are sporadic [29, 30]. In the UK, for example, formal recommendations or guidelines are lacking.

In this article, we review the clinical evidence for screening in other “high risk” conditions not included in the current UK guidance and discuss the health economic issues to be taken into consideration when considering CRC screening in these groups.

## Measuring risk

A major consideration in making decisions about which groups might benefit (or otherwise) from colonoscopy is the risk of CRC in that group. Risk can be expressed in different ways, as shown in Table 1.

**Table 1:** Definitions and considerations of different measures of disease occurrence

Term	Definition
Incidence	The number of new cases in a population over a defined time period.
(Point) prevalence	The number of cases in a population at a given time.
Risks and rates	Risk is a measure of disease occurrence (e.g. CRC) over a defined period of time, expressed as a proportion of the people at risk of the disease at the start of the time period. Rates take into account the time that *each person* was at risk of the disease; it is the disease occurrence over a defined time period divided by the sum of the time experience by everyone at risk of the disease.
Absolute measures of risk	The absolute risk is the (cumulative) probability of an event occurring over a defined period of time (e.g. 2 per 1000 people per year, or 7% over a lifetime). In terms of CRC, in many of the groups we consider in this article, the probability of disease is small and may have been estimated based on a small number of CRCs. This means that estimates of absolute risk may be imprecise, so it is important to pay attention to the uncertainty in the estimate of risk; this is reflected in the 95% confidence intervals.
Relative measures	These represent the risk or rate of an event in one group of people compared to another. In interpreting relative measures, it is important to bear in mind that very different clinical scenarios in terms of absolute risk (e.g. one where the absolute risk of disease is very small and another where it is large) can result in the same relative risk [31]. Standardized incidence ratio (SIR) expresses the rate of disease in the group of interest compared to the general population, taking account of the age and sex distribution of the group of interest. Relative risk/rate or risk/rate ratio (RR) expresses the risk (or rate) of disease in an exposed group compared to the risk (or rate) of disease in an unexposed group. It is the incidence (risk or rate) of disease in the exposed group compared to the incidence (risk or rate) of disease in the unexposed group. Odds ratio (OR) expresses the association between exposure and risk of disease. It is the odds of developing disease in the exposed group compared to the odds of developing disease in the unexposed group. Hazard ratio (HR) is the chance (hazard) of a particular event occurring in one group of people compared to another group, over time. It incorporates, for each person, the time “survived” from a defined starting point to the specific event (e.g. death from the disease). It is used when researchers are interested in the time it takes for an event to occur (e.g. survival analyses).

Table 2 outlines the risks of CRC for some of the conditions discussed in this manuscript and Table 3 outlines the risk in some of the current populations receiving CRC screening by colonoscopy, this is to give an indication of the risks in these populations for comparison with the areas under discussion. Table 4 provides examples of CRC screening recommendations.

**Table 2:**
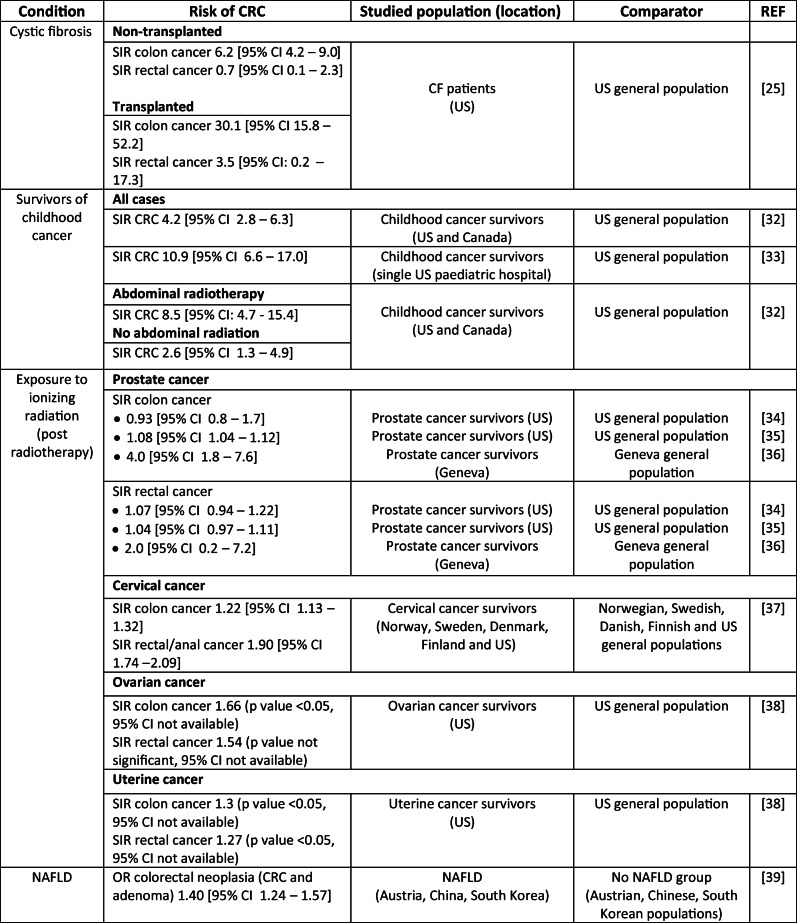
Risk of CRC per condition

**Table 3:**
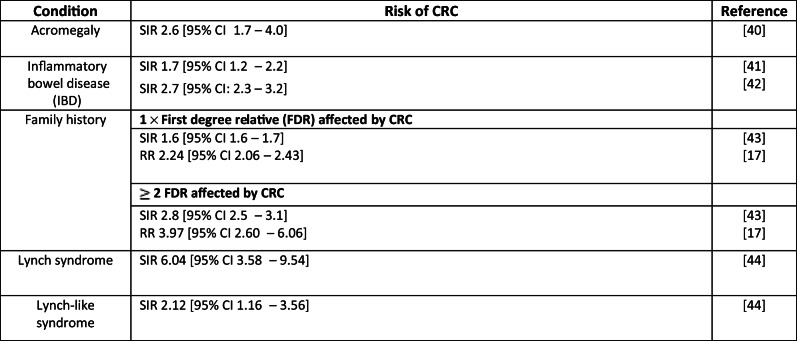
Examples of CRC risk in conditions considered in current screening

**Table 4:**
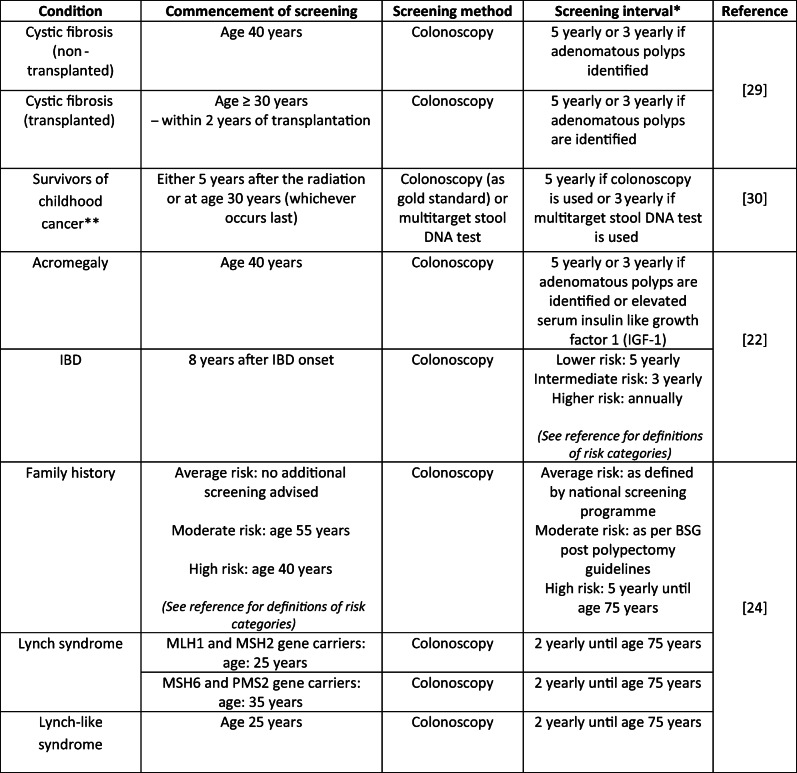
Examples of CRC screening recommendations

*Unless a shortened interval is indicated by individual findings

**Survivors of childhood cancer who have undergone radiation treatment to the abdomen, pelvis, spine (lumbar, sacral or whole) or total body irradiation

## Cystic fibrosis

Cystic fibrosis (CF) is an inherited genetic disorder caused by defects of the cystic fibrosis transmembrane conductance regulator gene. This leads to the production of abnormal secretions that affect the respiratory, gastrointestinal and reproductive systems.

In Europe, it is estimated that there are at least 48,000 patients living with CF, and almost 10,500 of these live in the UK [45].

Advances in the management of CF have led to a dramatic increase in life expectancy. In 2002, the mean age at death was 27 years whereas by 2018 the median predicted survival age in the UK was 47 years and rising [46, 47]. Data analyses from the UK CF Registry predict that 50% of patients living with CF aged 30 are now expected to live beyond the age of 55 years [48].

The increasing life expectancy of patients with CF has been accompanied by new challenges associated with older age, including the risk of malignancies such as CRC [49]. An increased risk of colon cancer has been observed in both transplanted and non-transplanted CF patients.

A 20-year nationwide study of 41,118 CF patients from the United States (US) identified SIRs of 6.2 [95% CI 4.2–9.0; *n* = 26 cancers] and 30.1 [95% CI 15.8–52.2; *n* = 11 cancers] for colon cancer in non-transplanted (cancers occurring before transplantation for transplanted patients included in this group) and transplanted patients respectively. The SIR for rectal cancer was not raised among non-transplanted patients (0.7 [95% CI 0.1–2.3; *n* = 2 cancers]); among those who were transplanted, it was increased 3-fold but did not reach statistical significance (3.5 [95% CI 0.2–17.3; *n* = 1 cancer]). [25]

A meta-analysis including data from transplanted and non-transplanted patients from both European and US populations including the study described above reported a pooled SIR of 10.91 [95% CI 8.42–14.11] for colon cancer [50•].

There is also evidence to suggest that adenoma formation (the usual precursor to colorectal cancer) occurs at a younger age and in an accelerated manner in CF patients. This is supported by the high proportion of patients that have adenomas on initial screening also having adenomas on subsequent procedures performed at short intervals [49]. However data on the value of CRC screening and surveillance in CF is limited.

In February 2018, the United States Cystic Fibrosis Colorectal Cancer Screening Task Force published recommendations for CRC screening in patients with CF (shown below), which were reviewed and endorsed by the American Gastroenterological Association (AGA) [29].

Recommendation statements:

1. The CF Foundation recommends that all decisions on colorectal cancer screening and surveillance in individuals with CF be based on shared decisions between the provider and individual with CF about treatment, comorbidities, safety and quality of life.
2. The CF Foundation recommends that all colorectal cancer screening and surveillance for individuals with CF are jointly managed by CF health care professionals and an endoscopist.
3. The CF Foundation recommends colonoscopy as the screening examination for CRC in individuals with CF.
4. The CF Foundation concludes that the evidence is insufficient to recommend the use of computed tomography colonography, stool-based tests or flexible sigmoidoscopy in individuals with CF for the purpose of CRC screening.
5. The CF Foundation recommends that CRC screening begin at age 40 years in individuals with CF with continued rescreening every 5 years.
6. The CF Foundation recommends that individuals with CF who have undergone a colonoscopy that had any adenomatous polyps have surveillance colonoscopy in 3 years, unless a shorter interval is indicated by individual findings, with subsequent intervals based on the most recent endoscopic examination.
7. The CF Foundation recommends that individuals with CF who are 30 years of age and older and have adequately recovered after receiving a solid organ transplantation begin CRC screening within 2 years of transplantation, except when they have had a negative colonoscopy within the past 5 years.
8. The CF Foundation recommends continued CRC rescreening every 5 years in individuals with CF who have received a solid organ transplant.
9. The CF Foundation recommends that individuals with CF who have undergone a solid organ transplantation and had colonoscopy that had any adenomatous polyps have surveillance colonoscopy in 3 years, unless a shorter interval is indicated by individual findings, with subsequent intervals based on the most recent endoscopic examination.
10. The CF Foundation recommends that adults with CF undergoing a colonoscopy receive intensive regimens for bowel preparation to allow for optimal examination. The intensive regimen should include 3–4 washes (minimum of 1 L purgative per wash) with the last wash occurring within 4–6 h before the examination

### Clinical evidence summary

Patients with CF, particularly those who have had a transplant, have a much higher risk of colon cancer than the general population. The evidence is unclear in relation to the risk of rectal cancer.

## Survivors of childhood cancer

Advances in treatment mean that survival rates for many childhood cancers have increased dramatically over the past 50 years. This means that the population of childhood cancer survivors is growing; there are currently estimated to be between 300,000 and 500,000 childhood cancer survivors in Europe and more than 35,000 in the UK [51, 52].

Survivors are at increased risk of developing subsequent malignant neoplasms (SMN) largely as a result of their cancer treatment, and there is evidence that these individuals develop gastrointestinal (GI) cancer more frequently and at a younger age compared to the general population [53, 54].

In a US study of 14,337 survivors of childhood cancer (index cancer diagnosed aged < 21 years), 802 SMNs were identified, of which 45 were GI in origin. The median age at diagnosis with GI cancer was 33.5 years, with a median time from index cancer diagnosis to GI SMN of 22.8 years [32•].

In the British Childhood Cancer Survivor Study, 17,981 survivors of childhood cancer (index cancer diagnosed aged < 15 years) were followed up for a median of 24.3 years. This study observed 1354 subsequent primary neoplasms, of which 105 were GI in origin. The observed number of GI SMNs in these patients was almost 5 times greater than would be expected in the general population, as shown by a SIR of 4.8 [95% CI 3.8–5.6] [54].

Compared to the general population, the risk of developing CRC is more pronounced among childhood cancer survivors who received abdominopelvic radiotherapy for their primary cancer [SIR 8.5, 95% CI 4.7–15.4], although a more modest increased risk is also observed in those who did not receive abdominopelvic radiotherapy [SIR 2.6, 95% CI 1.3–4.9] [32•].

There is evidence that the risk of CRC following radiotherapy is related to the dose received, with one study reporting that for every 10 Gy increase in dose to the colon, the risk of developing a subsequent CRC increased by 70% [OR 1.7, 95% CI 1.2–2.5] [33].

In addition to the risk associated with radiotherapy, exposure to alkylating agents used as part of chemotherapy regimens, for example procarbazine and platinum, have also been associated with an increased risk of GI SMNs [53].

Survivors of childhood cancer are also at higher risk of developing colorectal adenomas. A Dutch study of 6726 individuals (5843 5-year cancer survivors and 883 siblings) found a cumulative incidence of adenomas by age 45 years to be 3.6% [95% CI 2.2–5.6%] in those survivors who had radiotherapy to the colorectal area, 2% [95% CI 1.3–2.8%] in those survivors who did not receive radiotherapy and 1% [95% CI 0.3–2.6%] in the sibling control group [55].

A study of early colonoscopic screening in cancer survivors aged 35–49, who had received abdominal radiotherapy more than 10 years previously, found that the prevalence of adenomatous polyps in these patients (27.7%) was comparable to an average risk population aged 50 and older [56•].

The Scottish Intercollegiate Guidelines Network (SIGN) “Long Term follow up of survivors of Childhood Cancer” acknowledges the increased risk of subsequent primary cancers in this group and states that healthcare professionals should have an awareness of this increased risk; however, no specific guidance for screening/surveillance is given, other than to encourage participation in national screening programmes. [57]

In the USA however, the Children’s Oncology Group has produced long-term follow-up guidelines for survivors of childhood, adolescent and young adult cancers. It recommends that patients who have undergone radiation treatment to the abdomen, pelvis, spine (lumbar, sacral or whole) or total body irradiation should undergo screening and surveillance for CRC. The recommendation is that screening for CRC should begin either 5 years after the radiation or at age 30 years (whichever occurs last). The guidelines recommend colonoscopy as the gold standard for CRC screening; however, it is noted that multitarget stool DNA test is deemed a reasonable alternative, providing positive results are followed with a timely colonoscopy. Colonoscopy is advised every 5 years whereas if multitarget stool DNA test is used as the screening method, a 3-year interval is advised [30].

### Clinical evidence summary

There is evidence that childhood cancer survivors are at an increased risk of colorectal adenoma formation and CRC, particularly if they received abdominopelvic radiation treatment for the index cancer.

## Exposure to ionizing radiation (previous radiotherapy)

It is estimated that 2% of CRCs in the UK are attributable to past exposure to ionizing radiation [27]. The link between malignancy and exposure to ionizing radiation was initially observed in atomic bomb survivors after the World War 2 [58]. Radiotherapy (RT) for malignancy is now one of the most common reasons for patient exposure to ionizing radiation with approximately 50% of all patients with cancer receiving radiotherapy as part of the primary treatment regimen [59]. The latent period from radiation exposure to the development of radiation-induced malignancy is between 5 and 15 years [60].

During radiotherapy, high-dose ionizing radiation is delivered to the area of malignancy to induce cell death. Close proximity of organs means that radiation of healthy tissue is often unavoidable. During radiotherapy to pelvic malignancy (such as prostate and gynaecological cancers), parts of the colon and rectum are often within the field of irradiation.

### Prostate cancer

Rising incidence and survival rates mean that, in developed countries, there are more men living with prostate cancer than any other form of cancer; in the UK in 2020, there are estimated to be almost 420,000 prostate cancer survivors [61]. Incidence increases with age to peak in the 75–79 age group, and, on average, 87% of men diagnosed with prostate cancer are still alive after 5 years. Radiotherapy is given to around 30% patients as part of cancer treatment [62].

A meta-analysis of 719,823 patients with prostate cancer, reported in 9 studies, calculated a risk ratio (RR) of 1.33 [95% CI 1.10–1.67] for the development of rectal cancer in those who received radiotherapy treatment compared to those who did not. It should be noted that the incidence of subsequent rectal cancer was small in both irradiated and non-irradiated groups at 0.48% and 0.41% respectively [63]. The findings of another meta-analysis suggested that the association may be limited to those who had external beam radiotherapy (rather than brachytherapy), but few studies had reported on patients who received brachytherapy [64].

In terms of comparisons with the general population, there is conflicting evidence on whether men who have had radiotherapy for prostate cancer have increased risk of CRC. Reported risk estimates vary considerably with some studies reporting SIRs for colon cancer of around 1 and others a SIR of 4.0 [95% CI 1.8–7.6] [34–36]. The reported risk of subsequent rectal cancer is also variable with SIRs of 1.04 [95% CI 0.97–1.11], 1.07 [95% CI 0.94–1.22] and 2.0 [95% CI 0.2–7.2] [34–36].

#### Summary

Among men with prostate cancer, those who had RT have increased risk of rectal cancer compared to those not treated with RT. However, evidence is inconsistent as to whether risk of CRC is significantly increased compared with general population.

### Gynaecological malignancies

#### Cervical cancer

Cervical cancer accounts for around 3% of all new cancer cases (in women) in Europe and for around 1.7% of cancers in women in the UK [1, 65]. The incidence rates are highest for females aged 30–34 years, and 61% of women survive at least 5 years. In excess of 35,000 women are thought to be living with cervical cancer in the UK. Around 40% of patients receive radiotherapy as part of their cancer treatment [65].

A study of 104,760 survivors of cervical cancer, using data from European and US cancer registries, with an average follow-up of 12.2 years, reported SIRs of 1.22 [95% CI 1.16–1.30] and 1.84 [95% CI 1.72–1.98] for colon and rectal/anal cancer respectively. When analysed by whether patients received radiotherapy or not, SIRs of 1.22 [95% CI 1.13–1.32] and 1.90 [95% CI 1.74–2.09] for colon and rectal/anal cancer respectively were observed in the patients who received radiotherapy and SIRs of 0.93 [95% CI 0.8–1.08] for colon cancer and 1.28 [95% CI 1.06–1.55] rectal/anal cancer in those who did not [37].

A meta-analysis of 173,413 patients also identified an increased risk of rectal cancer in women who receive radiotherapy with a RR of 1.61 (95% CI: 1.10–2.35) for the development of rectal cancer in women with cervical cancer who received radiotherapy compared to women with cervical cancer who did not. [63]

Further analyses also involving data from the US Surveillance, Epidemiology and End Results (SEER) registries suggest that the increased risk of CRC compared to the general population becomes more pronounced in patients 10 years or more after their initial cancer diagnosis. This is evidenced by SIRs of 1.24 and 1.97 [*p* values < 0.05, 95% CI not available] for colon and rectal cancers respectively, for patients 10 years or more after their initial cancer diagnosis, compared to SIRs of 1.16 and 1.13 (*p* values not significant, 95% CI not available) for patients less than 10 years after their initial cancer diagnosis. It should be noted that these figures take into account all treatment modalities, and when analysed by whether patients received radiotherapy or not, the increased risk (at 10 years or more after initial cancer diagnosis) was only observed in those who received radiotherapy. SIRs of 1.43 and 2.78 (*p* values < 0.05, 95% CI not available) were observed for colon and rectal cancer respectively in those more than 10 years after their initial cancer diagnosis who received radiotherapy compared to SIRs of 0.98 and 0.99 (*p* values not significant, 95% CI not available) in those who did not [38].

##### Clinical evidence summary

Among survivors of cervical cancer, those who had RT have an increased risk of rectal cancer compared to those not treated with RT. Those treated with RT have an increased risk of subsequent CRC compared to the general population. This increased risk becomes apparent 10 years or more after the initial cancer diagnosis.

#### Ovarian cancer

Ovarian cancer accounts for 3.7% of all new cancer cases (in women) in Europe and 4% in the UK [1, 66]. The peak incidence rate is in women aged 75–79 years, and around 43% survive for 5 years or more after a diagnosis of ovarian cancer. It is estimated that there are slightly over 40,000 women living in the UK with a diagnosis of ovarian cancer. Only 2% of women diagnosed with ovarian cancer have radiotherapy as part of their primary cancer treatment [66].

Data from the SEER registries suggest that there is an overall increased risk of developing CRC following a diagnosis of ovarian cancer with SIRs of 1.26, 1.47 and 1.46 (*p* values < 0.05, 95% CI not available) for colon, rectosigmoid junction (including rectum) and rectal (rectum only) cancers respectively. In those patients diagnosed aged < 50 years, the risks were more pronounced: SIRs of 3.09, 2.77 and 2.65 (*p* values < 0.05 95% CI not available) for colon, rectosigmoid junction (including rectum) and rectal (rectum only) cancers respectively [38].

In relation to radiotherapy treatment, the SIR for colon cancer was 1.66 (*p* value < 0.05, 95% CI not available) for those who had radiotherapy and 1.21 (*p* value < 0.05, 95% CI not available) for those who had not. The increased risk in the radiotherapy group became significant > 10 years after the initial cancer diagnosis [38].

The increased risk of developing rectal cancer (including rectosigmoid junction) was not statistically significant in the radiotherapy group although the SIRs were slightly increased at 1.22 and 1.54 (95% CI not available) for rectosigmoid junction (including rectum) and rectal (rectum only) cancers respectively. However, this was based on only 3462 patients. The SIRs for these sites (rectosigmoid junction (including rectum) and rectal (rectum only)) were deemed statistically significant at 1.50 and 1.45 (*p* values < 0.05, 95% CI not available) respectively, for patients who did not receive radiotherapy, but in view of the low numbers of observed cancers, these data need to be interpreted with caution [38].

Hereditary conditions such as Lynch syndrome (hereditary non-polyposis colorectal cancer—HNPCC), where there is an increased risk of developing both CRC and ovarian cancer, may contribute to these figures; however, it should be noted that only around 2% of ovarian cancers are associated with Lynch syndrome [67]. This does increase to around 4% when stratified by the age of ovarian cancer diagnosis to < 40 years [68]. Patients with Lynch and related syndromes should be screened as per specific guidelines for example the BSG management of hereditary cancers guidelines [24].

##### Clinical evidence summary

Women with ovarian cancer have a modestly increased risk of developing CRC compared to the general population, and the risk is more pronounced in those diagnosed at a younger age. The risk of developing colon cancer is increased in those women who received radiotherapy. The effect of radiotherapy treatment on the development of rectal cancer is unclear.

#### Uterine cancer

Uterine cancer accounts for 6.6% of all new cancer cases (in women) in Europe and 5% in the UK [1, 69]. The incidence rates are highest for women aged 75–79 years. Seventy-six percent of patients survive for 5 years or more. An estimated 70,000 women are living with uterine cancer in the UK. Around 20% of patients receive radiotherapy as part of their primary cancer treatment [69].

Data from the SEER registries provide evidence of an increased risk of developing CRC following radiotherapy for uterine cancer. The SIRs for the development of colon and rectal cancer in those patients who received radiotherapy were 1.30 and 1.27 (*p* values < 0.05, 95% CI not available) respectively compared to 0.98 and 1.01 (*p* values not significant, 95% CI not available) in those who did not receive radiotherapy. The increased risk of CRC following radiotherapy becomes more apparent 10 years after initial cancer diagnosis [38].

Similarly, to what has been observed in ovarian cancer, those patients diagnosed with cancer aged < 50 years have a significantly increased risk of developing a subsequent CRC (taking all treatment modalities into account) compared to the general population, with SIRs of 3.64 and 3.38 (*p* values < 0.05, 95% CI not available) for colon and rectal cancers [38]. Again, hereditary conditions such as Lynch syndrome, where there is an increased risk of developing colorectal and uterine cancers, may contribute to these figures; however, only 2–3% of endometrial (uterine) cancers are attributable to Lynch syndrome [70].

##### Clinical evidence summary

The risk of developing CRC is markedly increased in women diagnosed with uterine cancer aged less than 50 years compared to the risk in the general population. This increased risk appears to be confined to women who received radiotherapy. This increased risk becomes apparent 10 years after the initial diagnosis.

## Non-alcoholic fatty liver disease

Non-alcoholic fatty liver disease (NAFLD) is a spectrum of liver disease ranging from steatosis, non-alcoholic steatohepatitis (NASH) to fibrosis and cirrhosis. It is the commonest cause of abnormal liver function tests (LFTs) in the UK, with up to 30% of the population estimated to have hepatic steatosis [71].

The association between NAFLD and colorectal neoplasia has predominantly been studied in Asian populations. A meta-analysis of 91,124 asymptomatic individuals (which included 29,319 individuals with NAFLD) who underwent screening colonoscopy found the overall cumulative prevalence for colorectal adenomas to be 20.4% [95% CI 19.9–20.9] in patients with NAFLD and 15.8% in those without [95% CI 15.5–16.1]. CRC rates were 2.4% [95% CI 2.2–2.6] and 1.97% [95% CI: 1.9–2.0] in those with and without NAFLD respectively. Patients with NAFLD had a 1.4 times higher risk of colorectal neoplasia compared to those without [39].

The severity of the liver disease has been shown to be associated with the risk of colorectal neoplasia; however, further work with larger studies is needed to further establish this and verify whether the same association is evident in non-Asian populations [28].

### Clinical evidence summary

There is limited evidence in this area; further research is needed to clarify whether there is any increased risk in this group.

## Health economics considerations

Economic aims are extremely important across the healthcare sector where resources are scarce and even more important in areas that have capacity issues, such as endoscopy services. It is vital that any decision regarding screening and monitoring of groups of the population is underpinned by the economic principles that ensure efficiency and maximize the health benefit realized.

Whilst there is a strong body of economic evidence to support national population-based screening programmes, there is limited relevant economic evidence to support screening of the high-risk groups outlined above [72]. Full review of the economic evidence is beyond the scope of this paper.

The benefits of screening include prevention of CRC or early diagnosis of cancer, which can mean less complex, less costly treatments that achieve longer survival and better quality of life for the individuals. As there is evidence to suggest that the CRC risk in these groups (or, at least, some subgroups) is generally higher than that of the general population, there are economic arguments to consider an alternative approach to screening and surveillance.

Any economic evaluation would need to consider a number of different costs, including the economic costs to the health services associated with the provision of the screening tests and any subsequent tests, treatments and surveillance that are required as a result of screening. These include the costs of harms that may occur as a result of screening, such as a complication following colonoscopy. The costs to the patient also need to be considered such as the financial costs to individuals (including lost income from time away from work) and psychological costs, for example anxiety and the impact on quality of life associated with screening.

A study assessing the psychological impact of participation in CRC screening was undertaken within the Australian population-based CRC screening programme. The study identified that individuals who received a positive FIT result (FIT used as initial screening test and colonoscopy subsequently performed in those with positive FIT result) experienced a higher state of anxiety compared to those with a negative result. Although this level of anxiety improved over time (measured 1 year after notification of the FIT result), it still remained higher than in those who received a negative FIT result [73]. This highlights a potential longer term, psychological impact to patients that needs to be acknowledged when considering the risks and benefits of screening.

Further, there is a need to consider the implementation and capacity issues around any such programme. Any evaluation in the area will be limited by the knowledge that we have around the natural progression of the disease and the uncertainty around some of the sub-group risk profiles. However, these uncertainties are inherent in many economic evaluations and can be quantified and considered as part of the analysis. These same uncertainties are clearly inherent in the decisions made that are not based on a transparent evaluation. Undertaking analyses for the separate groups and scenarios will maximize the benefit from screening and monitoring within the capacity of current services, whilst also allowing for an explicit consideration of the cost and benefits trade off should it be possible to increase current capacity.

## Discussion

In addition to the broader known risk factors, there is evidence that there are a range of conditions and treatments placing patients at a higher risk of developing CRC, and generally, these are not included in current screening and surveillance guidance. This review of the literature indicates that the most compelling evidence for undertaking screening is for patients with CF and—to a lesser extent—survivors of childhood cancer.

The risk of developing CRC for a patient with CF is around 6–30 times that of the general population depending on their transplant status, and the frequency of CRC in survivors of childhood cancer is between 4 and 11 times that of the general population. The relatively low prevalence of these conditions means that the additional burden on endoscopy services would not be that vast. Recommendations for screening in these patient groups are already in place in the USA [29]. These recommendations are well considered and emphasize the importance of shared decision-making with patients. A full health economics evaluation based on the relevant healthcare system would be important if adoption of screening in other settings was to be considered.

The life expectancy of an individual and the latency period from an exposure to the development of a malignancy are key considerations in assessing the suitability of screening. The latent period from radiation exposure to the development of radiation-induced malignancy is between 5 and 15 years [60]. In survivors of childhood cancers, their life expectancy is likely to be sufficient to benefit from screening even when taking the latency period into account. It remains uncertain, however, what screening should be involved (e.g. starting age, screening interval) to provide the best balance of benefits and cost. A health economic evaluation might usefully compare different scenarios to inform decision-making.

In relation to those groups who have received radiotherapy for pelvic malignancies, there is an increased risk of CRC in patients with cervical, ovarian and uterine cancers who received radiotherapy, compared to the general population. The evidence of the risk of CRC in men with prostate cancer treated with radiotherapy is inconsistent.

In relation to cervical cancer, the peak incidence is aged 30–34 years, and similarly to survivors of childhood cancers, their life expectancy is likely to be sufficient to benefit from screening even when taking the latency period from radiation exposure to the development of malignancy into account.

The peak incidence rates for prostate, ovarian and uterine cancer occur at an older age, and when combined with the latency period from exposure to radiation before the development of further malignancy, there may not be a significant benefit of CRC screening in these patients. The BSG guidance for post-polypectomy and post-cancer resection surveillance does not recommend surveillance in patients with life expectancy < 10 years or if older than around 75 years [23]. It is worth noting that due to the peak incidence rates for these cancers occurring at an older age, a number of these patients will be eligible to participate in population-based screening programmes; however, special consideration needs to be given to those diagnosed with cancer aged < 50 years (i.e. more than 10 years outside the eligibility for the UK’s population-based screening programme).

We acknowledge that radiation therapy techniques have developed rapidly over the past two decades, and more targeted approaches to radiotherapy are now routinely used [74]. This may have implications for the future risk of CRC in current patients undergoing radiotherapy. It is also important to note that there are different types of radiotherapy including external beam radiotherapy, total body irradiation and brachytherapy, and many of the relevant studies do not present their data by which specific radiotherapy was used but rather by whether radiotherapy was received or not. Understanding remains limited, therefore, on whether risk of CRC differs by specific therapy (or indeed specific courses).

In survivors of childhood cancers and ovarian cancer, the risk of developing CRC compared to the general population was increased both in those that received radiotherapy and, to a lesser extent, those who did not. The increase in the non-radiotherapy groups may be explained by the effects of other treatments these patients receive or by shared risk factors for both the index cancer and subsequent cancers. Further research to better understand this would be of value.

Although there is evidence that the presence of NAFLD increases the risk of adenomas and CRC, this has not been demonstrated in a Western population. In addition to this, it is not known whether the presence of NAFLD itself increases the risk of colorectal neoplasia or whether it is related to shared metabolic risk factors between NAFLD and CRC. NAFLD has a high prevalence in the UK, and if CRC screening was to be adopted, this would place a significant demand on endoscopic services. Further work is needed in this field to support the consideration of CRC screening in these patients.

Alongside consideration of the specific conditions which may warrant enhanced screening, there are important logistical issues to consider if systematic screening/surveillance was to be offered to high-risk groups. Any organized screening programme needs to both define the target population and be able to identify those who comprise that target population [75]. This means that a complete and up-to-date register of people in the target population is a pre-requisite for effective call-recall based screening. Creating and maintaining such registers would not be a trivial consideration in many healthcare systems.

Colonoscopy is currently the investigation offered for screening of high-risk groups and is the most widely studied. However, there are already considerable pressures upon endoscopy services. Consideration of expansion of screening to high-risk groups must, therefore, consider the role of biomarkers as part of a screening strategy. Reviewing the role of biomarkers is beyond the scope of this paper, but the role of FIT and other biomarkers should be considered, for example, in the UK, FIT has been used as a means of triaging Lynch syndrome patients during the COVID-19 pandemic [76]. The development of risk prediction models may change the way CRC screening is performed more widely and be used to deliver more intelligent screening at the level of an individual.

## Conclusions

There are a number of conditions, not historically covered by screening programmes, which place individuals at significantly increased risk of colorectal cancer. Screening appears to be justified in a number of these settings and should be considered based upon ability to implement such a programme and involve a thorough health economic evaluation.
